# Expression patterns of *prdm1* during chicken embryonic and germline development

**DOI:** 10.1007/s00441-014-1804-1

**Published:** 2014-04-02

**Authors:** Zhiyi Wan, Lei Rui, Zandong Li

**Affiliations:** State Key Laboratory for Agrobiotechnology, College of Biological Sciences, China Agricultural University, No.2 Yuan Ming Yuan West Road, Beijing, 100193 People’s Republic of China

**Keywords:** PRDM1, Gene expression, Embryonic development, Primordial germ cells, Blastodermal cells, Chicken (White Leghorn)

## Abstract

PRDM1 (PR domain containing 1) is a transcriptional repressor that has been identified in various species and is crucial for cell growth, differentiation and development. However, the expression pattern and role of PRDM1 in development has not been sufficiently established in birds. We therefore investigate the spatio-temporal expression of PRDM1 in various tissues, especially in the germline, during chicken development, providing the basis for functional study. Our results show that *prdm1* mRNA was expressed in blastodermal cells (BCs) at stage X and in various tissues including the liver, skin, lung, kidney, eye, bursa of fabricius, spleen, proventriculus, gizzard, intestine, testis, ovary, tongue, feathers and thymus but was not or was only sparcely present in the heart, brain and skeletal muscle. The level of *prdm1* mRNA was highest in the BCs among all tissues tested and significantly changed during development in many tissues, such as the blastoderm, bursa of fabricius, spleen, feathers and germline. Furthermore, the expression of the PRDM1 protein generally paralleled the mRNA results, except for in the gizzard. Immunohistochemistry also revealed that PRDM1 was localized in the smooth muscle. In addition, during germline development, PRDM1 was found to be continuously expressed in the presumptive primordial germ cells (PGCs) at stage X, the circulating PGCs in blood and the germ cells in the gonads from embryonic day 6 to adult in both males and females. The expression pattern of PRDM1 in chicken thus suggests that this protein plays an important role during chicken development, such as in BC differentiation, feather formation and germ cell specification.

**Electronic supplementary material:**

The online version of this article (doi:10.1007/s00441-014-1804-1) contains supplementary material, which is available to authorized users.

## Introduction

PR domain containing 1 (also known as PRDM1 and BLIMP-1) is a transcriptional repressor that contains five Kruppel-type zinc fingers and a PR/SET domain and that binds to a specific DNA sequence and recruits epigenetic modifiers (Bikoff et al. 2009). It is a member of the PRDM gene family, which contains 16–17 family members in mammals (Fumasoni et al. 2007). In humans, PRDI-BF1, a homolog of PRDM1, was first identified as a repressor of interferon-β gene expression (Keller and Maniatis 1991). It was later determined to program the terminal differentiation of B cells into immunoglobulin-secreting plasma cells (Turner et al. 1994). PRDM1 has also been detected in a subset of diffuse large B cell lymphomas (Pasqualucci et al. 2006), macrophages (Chang et al. 2000) and natural killer cells (Smith et al. 2010). In addition, recent studies have implied that PRDM1 functions in regulating T cell activation and homeostasis (Martins et al. 2006). However, the involvement of PRDM1 is not restricted to the immune system, as it has recently been shown to be crucial for cell growth, differentiation and development through its diverse molecular partnerships.

For example, in mouse, PRDM1 is widely expressed in diverse endodermal and mesodermal tissues (Chang and Calame 2002). PRDM1-deficient embryos die at mid-gestation but early axis formation proceeds normally (Vincent et al. 2005). In addition, PRDM1 regulates the development of the posterior forelimb, caudal pharyngeal arches, heart and sensory vibrissae (Robertson et al. 2007). It also controls the pattern of gene expression in epidermal keratinocytes (Magnusdottir et al. 2007) and the sebaceous gland (Horsley et al. 2006). No defects in either muscle or neural crest lineages have been reported in the absence of PRDM1. In contrast, in zebrafish, PRDM1 is not required for germ cell specification but is required for the specification of slow-twitch muscle fibers (Baxendale et al. 2004), fin bud outgrowth (Wilm and Solnica-Krezel 2005) and the common progenitors of the neural crest and sensory neurons (Hernandez-Lagunas et al. 2005; Roy and Ng 2004). In *Xenopus* and sea urchin, the *prdm1* homologs *Xblimp1* and *SpKrox1*, respectively, are also required during early embryonic development (de Souza et al. 1999; Wang et al. 1996).

Notably, PRDM1 has also been detected in the migrating primordial germ cells (PGCs) in mouse (Chang and Calame 2002) and PRDM1 deficiency results in a complete lack of PGCs (Vincent et al. 2005). In particular, in a few of the most proximal posterior epiblast cells at around embryonic day (E) 6.25, PRDM1 and PRDM14, as key factors for germ cell specification (Ohinata et al. 2005; Yamaji et al. 2008), are independently activated by bone morphogenetic protein (BMP) signals from the extra-embryonic ectoderm and these cells increase in number and go on to form PGCs via the integration of three key events: repression of the somatic program (Saitou et al. 2002), reacquisition of potential pluripotency (Yabuta et al. 2006) and genome-wide epigenetic reprogramming (Seki et al. 2007). PRDM1 is critical for all three of these events, whereas PRDM14 ensures the last-mentioned two (Yamaji et al. 2008).

Although the function of PRDM1 in embryonic development and organogenesis has been reported in many species, little is known about its role in birds. Its homolog in chicken, *cBlimp1*, has been cloned and detected in the apical ectodermal ridge and posterior dorsal ectoderm of the developing limb buds, developing eyes, branchial arches and otic placodes by in situ hybridization (Ha and Riddle 2003). cBLIMP1 is a highly conserved protein, exhibiting 77 % identity in its amino acid sequence between mice, human and chicken homologs and 81 % identity between chicken and human homologs (Ha and Riddle 2003). A recent study has demonstrated that PRDM1 is expressed in all differentiated muscle cells in chicken (Beermann et al. 2010).

The crucial role of PRDM1 in mice PGCs suggests the existence of a similar role in chicken PGCs. Chicken PGCs originate from the epiblast (Eyal-Giladi et al. 1981), circulate through the vascular system and then settle in the gonadal anlagen (Fujimoto et al. 1976). Germ cells are then thought to arise through a gradual epigenetic process beginning at around stage X as a result of their close association with the extra-embryonic mesoderm (Ginsburg 1997; Karagenc et al. 1996). However, the isolation of the chicken *vasa* homolog (*Cvh*) gene and the subcellular distribution of the CVH protein suggest that the chicken germ lineage is maternally predetermined (Tsunekawa et al. 2000). However, functional studies have yet to be conducted. *Prdm1* mRNA has been detected in PGCs in the chicken (Motono et al. 2008) but its role with respect to PGCs has not yet been reported.

Chicken embryos are an ideal model for studying developmental biology and have been widely utilized to analyze the expression pattern of candidate genes at specific stages. Here, we describe our detailed investigation of the spatio-temporal expression of PRDM1 in various tissues, especially in the germline, during chicken development and speculate its possible roles in the chicken with respect to its known roles in other species.

## Materials and methods

### Experimental animals and animal care

Fertilized White Leghorn chicken eggs (*Gallus gallus*) were purchased from the Experimental Station of China Agricultural University (Beijing, China) and incubated blunt-end-up at 37.5 °C under a relative humidity of 55-65 % with 90° tilting once every hour (P-008B Biotype, Showa Furanki, Saitama, Japan). The resultant embryos were staged according to Hamburger and Hamilton (1951) or Eyal-Giladi and Kochav (1976). Animal welfare and experimental procedures adhered to the Institutional Guidelines of the Care and Use of Laboratory Animals at China Agricultural University (Beijing, China).

### Sample collection

Intrauterine embryos were obtained from anesthetized hens according to a previous report (Eyal-Giladi and Kochav 1976). Blastodermal cells (BCs) were obtained from the blastoderms at stages V (uterine age of 8–9 h), VII (uterine age of 12–14 h) and X (freshly laid egg; uterine age of ∼20 h) by using filter paper rings as described previously (Gao et al. 2011). Whole embryos were collected at stage 6 (E1), stage 12 (E2) and stage 18 (E3). Blood samples including PGCs were collected at stages 13–15. Blood (2 μl) was obtained from each embryo through the vitelline artery by using a sharply cut, glass tip that was approximately 50 μm in diameter. Various tissues were dissected from E14 and post-hatching 1-day chickens. We collected embryonic gonads on E6, E7, E8, E10, E12, E14, E16 and E18, testes and ovaries at 1 day, 8 weeks and 25 weeks, the bursa of fabricius and spleens on E10, E12, E14, E16, E18, 1 day and 8 weeks and the skin and feathers on E10, E12, E14, E16, E18, 1 day and 25 weeks.

### Total RNA extraction and reverse transcription

Total RNA was extracted from the above samples by using a total RNA extraction kit (Tiangen, Beijing, China) or an RNeasy Plus Mini kit (Qiagen, Valencia, Calif., USA) according to the manufacturer’s protocol. All traces of DNA in the samples were removed with RQ1 RNase-Free DNase (Promega, Madison, Wis., USA) according to the manufacturer’s protocol prior to reverse transcription (RT). Approximately 1 μg oligo(dT)15-primed total RNA samples were reverse-transcribed with the Reverse Transcription System (Promega, Madison, Wis., USA) following the manufacturer’s instructions.

### Analysis by RT-polymerase chain reaction

The RT-polymerase chain reaction (RT-PCR) was performed to examine the tissue-specific expression of *prdm1* during embryonic development. The cDNAs from the blastoderms/embryos at stages X, 6, 12, 18 and 22 and the heart, liver, skin, lung, kidney, brain, eye, bursa of fabricius, spleen, proventriculus, gizzard, intestine, testis, ovary, skeletal muscle, tongue, feathers and thymus on E14 and 1 day were amplified with specific primers. The set of primers for *prdm1* included the forward primer TCATACCAGCACCTAACAGTGCCT and the reverse primer TCTTCAGTGGGTATGGGAGGGTTT, which spanned a splicing site and were designed to give a product of 240 bp. Chicken glyceraldehyde-3-phosphate dehydrogenase (*gapdh*) was used as a control with the forward primer CAGATCAGTTTCTATCAGC and reverse primer TGTGACTTCAATGGTGACA, which gave a product of 343 bp. Each 20-μl PCR mix contained 1 μl cDNA, 10 μl PCR buffer, 7 μl water and 10 pmol of each forward and reverse primer. The reaction was performed with an initial incubation at 94 °C for 5 min, followed by 30 cycles at 94 °C for 30 s, 64 °C for 30 s and 72 °C for 30 s. The reaction was terminated by a final incubation at 72 °C for 5 min.

### Quantitative RT-PCR analysis

We performed quantitative RT-PCR (qRT-PCR) to quantify the expression level of *prdm1* during embryonic development and the time-dependent expression of *prdm1* in the BCs, bursa of fabricius, spleens, skin, feathers and germline. The cDNAs from the blastoderms/embryos at stages V, VII, X, 6, 12 and 18, the gonads of male and female embryos on E6, E7, E8, E10, E12, E14, E16 and E18, the testes and ovaries of chickens at 1 day and 25 weeks, the bursa of fabricius and spleens on E10, E12, E14, E16 and E18 and at 1 day and 10 weeks and the skin and feathers on E10, E12, E14, E16 and E18 and at 1 day and 25 weeks were amplified with specific primers. The following primers were designed to amplify the chicken *prdm1* gene (fragment size: 205 bp): sense primer, ACACAGCGGAGAGAGACCAT; antisense primer, GCACAGCTTGCACTGGTAAG. Chicken *gapdh* was used as an internal control (fragment size: 123 bp): sense primer, TGCCATCACAGCCACACAGAAG; antisense primer, ACTTTCCCCACAGCCTTAGCAG.

qRT-PCR analysis was performed by using the LightCycler 480 system and the LightCycler 480 SYBR Green I Master kit (Roche, Mannheim, Germany) according to the manufacturer’s instructions. The thermal cycling parameters were as follows: 95 °C for 10 min, 40 cycles of 95 °C for 15 s and 60 °C for 1 min, followed by one cycle of 95 °C for 15 s, 60 °C for 15 s and 95 °C for 15 s. A final step was performed to obtain a melting curve for each PCR product to determine the specificity of amplification. The expression level of *prdm1* was calculated relative to the expression of the *gapdh* by the 2^-ΔΔCt^ method and was expressed as the fold change relative to the control sample. All standard dilutions and samples were analyzed in triplicate on the same plate and each reaction plate contained two standard curves for both the target and reference genes in the same preparation.

### Antibodies

To detect PRDM1 by Western blot and immunocytochemistry (IHC), we used the monoclonal rabbit anti-PRDM1 antibody from Cell Signaling Technology (CST, Danvers, Mass., USA) and the polyclonal rabbit anti-PRDM1 antibody from GenScript (Piscataway, N.J., USA). The polyclonal antibodies against CVH and chicken deleted in azoospermia-like (cDAZL) were kindly provided by Dr. Masa-aki Hattori (Kito et al. 2010). The mouse anti-stage-specific embryonic antigen-1 (SSEA-1) monoclonal antibody (MC-480) was from the Developmental Studies Hybridoma Bank (Iowa City, Iowa, USA). The mouse anti-GAPDH monoclonal antibody, the horseradish-peroxidase (HRP)-conjugated goat anti-rabbit IgG and the goat anti-mouse IgG were from CoWin (Beijing, China).

### Western blot

Tissues from chicks of 1 day of age were dissected and homogenized on ice in tissue protein extraction reagent (Boster, Wuhan, China) containing 1 mM phenylmethane sulfonyl fluoride. Approximately 40 g of the resultant protein was subjected to SDS-polyacrylamide gel electrophoresis in 10 % polyacrylamide gels and then electrotransferred onto Immobilon polyvinylidene difluoride membranes (Millipore, Bedford, Mass., USA). The membranes were blocked in phosphate-buffered saline (PBS) with 0.1 % Tween-20 (PBST) containing 5 % non-fat dry milk, incubated overnight at 4 °C with primary antibody for PRDM1 (1:1,000) or GAPDH (1:3000). The blots were washed three times with PBST for 10 min each and then incubated with the appropriate secondary antibody (1:3000; PRDM1: HRP-conjugated goat anti-rabbit IgG; GAPDH: HRP-conjugated goat anti-mouse IgG) for 1 h at room temperature. Following three 15-min washes with PBST, the blots were visualized on film by using SuperSignal West Pico Substrate (Thermo Scientific, Pittsburgh, Pa., USA).

### Immunocytochemistry

Tissues from distinct developmental stages were fixed in 4 % paraformaldehyde (PFA) overnight at 4 °C. The fixed samples were processed in an alcohol gradient and xylene and then embedded in paraffin prior to being cut into 7-μm sections for IHC analysis. IHC was performed with the StreptAvidin-Biotin-peroxidase complex kit (Boster, Wuhan, China) for most tissues and the anti-rabbit IgG-HRP kit (Boster, Wuhan, China) for the liver and kidney according to the manufacturer’s protocols. The sections were incubated with primary antibody at 1:250 (CST or Genscript) in a humidified chamber overnight at 4 °C. The immunoreactive sites were visualized by incubation with freshly prepared 3,3′-diaminobenzidine-tetrachloride. The sections were then counterstained with hematoxylin and examined under a light microscope (Olympus, Narishige, Japan). In the same tissue, negative controls were used to check for non-specific development and were treated as described above, except that non-immune serum was used instead of primary antibody.

### Immunofluorescence

The BCs and blood cells were dispersed onto glass slides and then fixed in 4 % PFA for 20 min at room temperature. The cells were washed three times with PBS for 3 min each, followed by permeabilization in PBS with 0.1 % Triton X-100 for 10 min at room temperature. Immunofluorescence (IF) was performed by using a StreptAvidin-Biotin-fluorescein-isothiocyanate complex kit and StreptAvidin-Biotin-Cy3 complex kit (Boster, Wuhan, China) according to the manufacturer’s protocol. Sections or cells were incubated with primary antibody, which was diluted as indicated: anti-PRDM1 antibody (1:250), anti-cDAZL antibody (1:100), anti-CVH antiserum (1:300) and anti-SSEA-1 antibody (1:500). For double-staining with a second primary antibody, the IF protocol (block, primary antibody and secondary antibody incubation) was performed sequentially. Sections or cells were counterstained with 4,6-diamidino-2-phenylindole (DAPI) dissolved in PBS and detected under a fluorescence microscope (Olympus, Narishige, Japan). Negative controls were treated as described above, except that non-immune serum was used instead of primary antibody.

### Periodic-acid-Schiff staining

Blood samples were fixed in Carnoy’s fluid for 20 min. After being rinsed with PBS, the cells were immersed in 0.5 % periodic acid (Sigma-Aldrich, St. Louis, Mo., USA) for 10 min, washed three times with PBS and subsequently treated with Schiff’s reagent (Sigma-Aldrich) for 10 min. The samples were then carefully washed three times with deionized H_2_O for 4 min each and then were observed under a light microscope (Olympus, Narishige, Japan). All procedures were performed at room temperature.

### Statistical analysis

The results are given as the mean ± standard error of the mean (SE). Statistically significant differences were computed by using the Kruskal-Wallis test or Student’s *t* test with the statistical software SPSS (Version 20.0). Probability (*P*) values of less than 0.05 were considered to be statistically significant.

## Results

### Expression of *prdm1* in early blastoderms/embryos and in various tissues

**Fig. 1:**
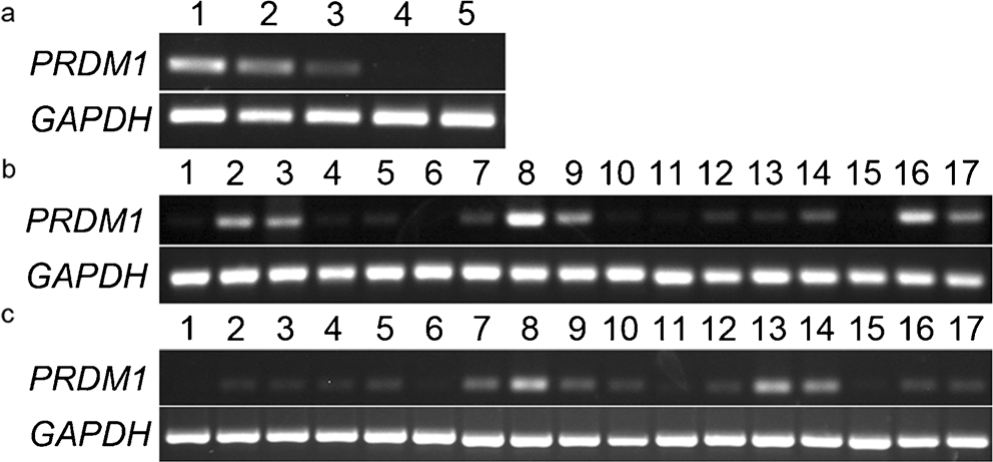
Expression of *prdm1* (*PR domain containing 1*; *PRDM1*) mRNA in early blastoderms/embryos and various tissues (*GAPDH* housekeeping gene *gapdh* as a reference). Bands with the expected size of the *prdm1* product (∼240 bp) were obtained from the samples as indicated. **a** Expression of *prdm1* in early blastoderms/embryos (*lanes 1–5* blastoderms/embryos at stage X, stage 6, stage 12, stage 18 and stage 24, respectively). **b** Expression of *prdm1* in various early tissues on E14 (*lanes 1–17* heart, liver, skin, lung, kidney, brain, eye, bursa of fabricius, spleen, proventriculus, gizzard, intestine, testis, ovary, skeletal muscle, tongue and feathers, respectively). **c** Expression of *prdm1* in various early tissues at 1 day (*lanes 1–17* heart, liver, skin, lung, kidney, brain, eye, bursa of fabricius, spleen, proventriculus, gizzard, intestine, testis, ovary, skeletal muscle, tongue and thymus, respectively)

The expression of *prdm1* in the early embryo at stages X, 6, 12 and 18 and in the heart, liver, skin, lung, kidney, brain, eye, bursa of fabricius, spleen, proventriculus, gizzard, intestine, testis, ovary, skeletal muscle, tongue, feathers and thymus on E14 and at 1 day was examined by RT-PCR. During early embryonic development, *prdm1* was detected at a high level in the embryo at stage X, decreased to a moderate level at stage 12 and was barely detected at stage 18 (Fig. 1a). Expression of *prdm1* was also detected in most embryonic tissues on E14 (Fig. 1b). Specifically, *prdm1* was detected at a strong level in the bursa of fabricius, skin, liver, spleen, tongue and feathers and at a low level in the lung, kidney, proventriculus and gizzard. However, it was not or was only barely detected in the heart, brain and skeletal muscle. The expression pattern at 1 day was similar to that on E14 except the feathers at 1 d was not investigated (Fig. 1c). Moreover, *prdm1* was observed in the thymus at 1 day (Fig. 1c). Sequencing was used to confirm the identity of the amplification product (not shown).

### Quantitative analysis of *prdm1* in BCs at stage X and tissues on E14 and at 1 day

**Fig. 2:**
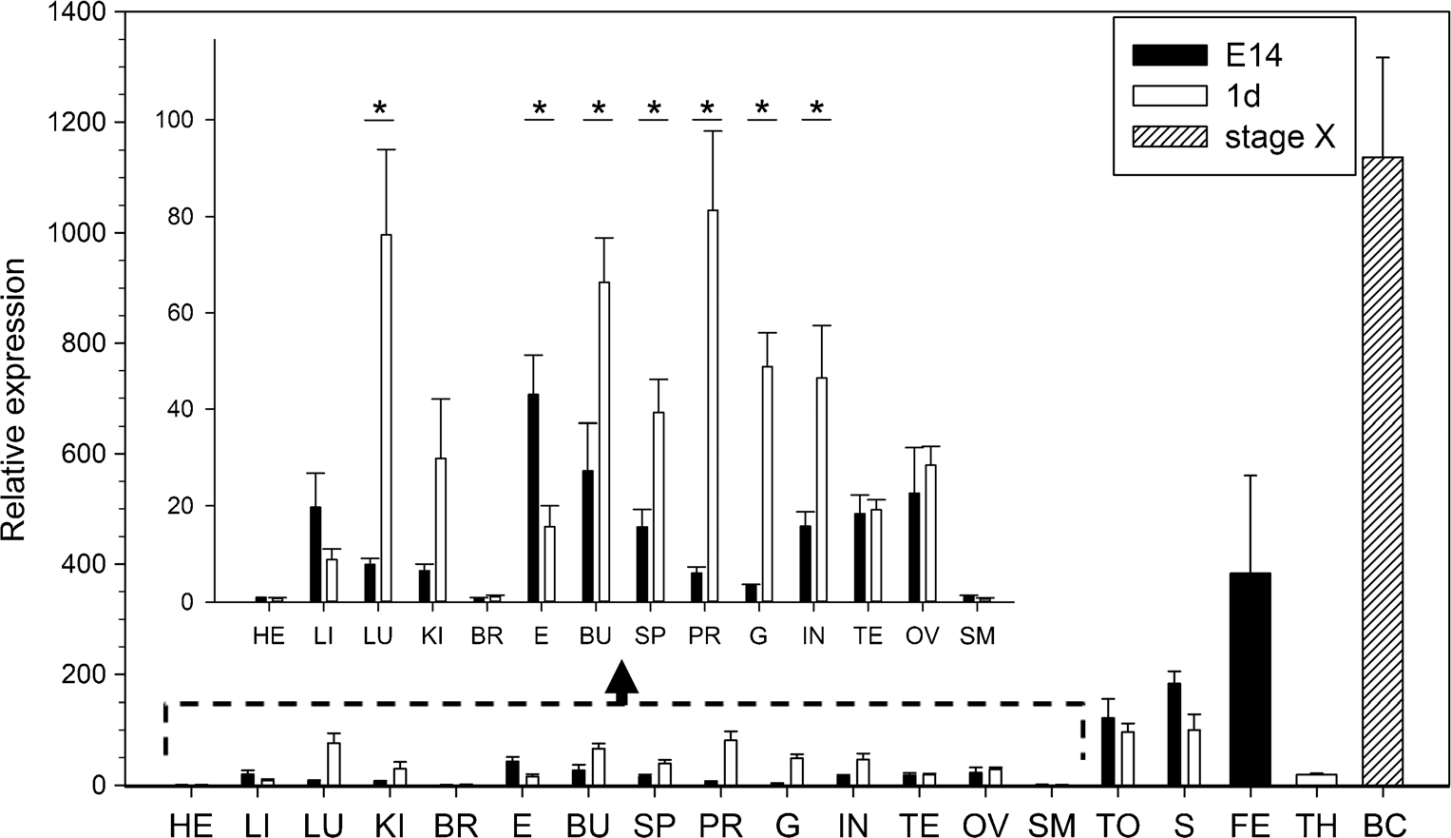
Quantitative reverse transcription with the polymerase chain reaction (RT-PCR) analysis of *prdm1* in BCs at stage X and in various tissues on embryonic day 14 (*E14*) and at 1 day (*1d*). The expression level of *prdm1* was calculated relative to the expression of *gapdh* by the 2^-ΔΔCt^ method and is expressed as the fold change relative to that in heart on E14. Data are expressed as the mean ± SE of five independent experiments. Statistically significant differences from the tissues on E14 and at 1 day were analyzed by Student’s *t* test; *P < 0.05 (*HE* heart, *LI* liver, *LU* lung, *KI* kidney, *BR* brain, *E* eye, *BU* bursa of fabricius, *SP* spleen, *PR* proventriculus, *G* gizzard, *IN* intestine, *TE* testis, *OV* ovary, *SM* skeletal muscle, *TO* tongue, *S* skin, *FE* feather, *TH* thymus, *BC* blastodermal cells)

To determine the relative expression levels of *prdm1* in various tissues, qRT-PCR analysis was performed with specific primer sets for *prdm1*, with *gapdh* being used as the reference housekeeping gene. Expression of *prdm1* was detected at a low level in all the analyzed samples relative to *gapdh* (Fig. 2). In particular, the expression level of *prdm1* was highest in the BCs at stage X (1137 ± 180-fold), followed in descending order by the feathers, skin, tongue and other tissue on E14 and at 1 day and was at its lowest level in the heart, brain and skeletal muscle (Fig. 2). The expression levels of *prdm1* in the lung, bursa of fabricius, spleen, proventriculus, gizzard and intestine at 1 day were significantly higher than those on E14. However, the expression level of *prdm1* in the eye at 1 day was significantly lower than that on E14 but no significant difference was seen in the other tissues between E14 and 1 day (Fig. 2).

### Quantitative analysis of *prdm1* in blastoderms/embryos during early embryonic development

**Fig. 3:**
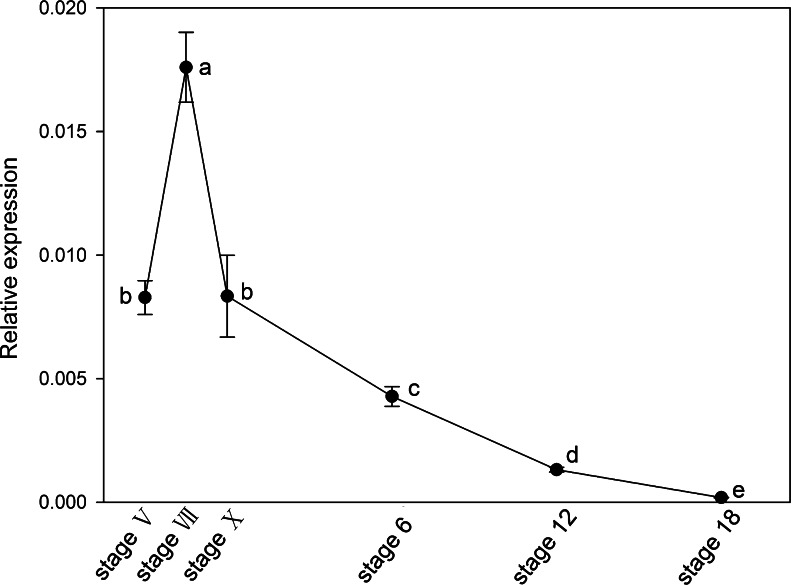
Quantitative RT-PCR analysis of *prdm1* in blastoderms/embryos during early embryonic development. cDNAs from blastodermal cells at stages V, VII, and X and from whole embryos at stages 6, 12, and 18 were amplified with *prdm1*-specific primers. The expression level of *prdm1* was calculated relative to the expression of *gapdh* by the 2^-ΔΔCt^ method. Data are expressed as the mean ± SE of three independent experiments. Values with the *same letter* do not differ significantly according to the Kruskal-Wallis test; *P* < 0.05

As we detected *prdm1* in the BCs at stage X, we next explored the relative abundance of *prdm1* from the cleavage stage to stage 18 by qRT-PCR. *Prdm1* expression was detected at a low level relative to *gapdh* from stages V to 18 (Fig. 3). *Prdm1* expression was highest at stage VII and then significantly decreased over the developmental course. No significant difference in *prdm1* expression was observed between stage V and stage X. It was not or just barely detected at stage 18 (Fig. 3).

### Quantitative analysis of *prdm1* during development of the bursa of fabricius and spleen

**Fig. 4:**
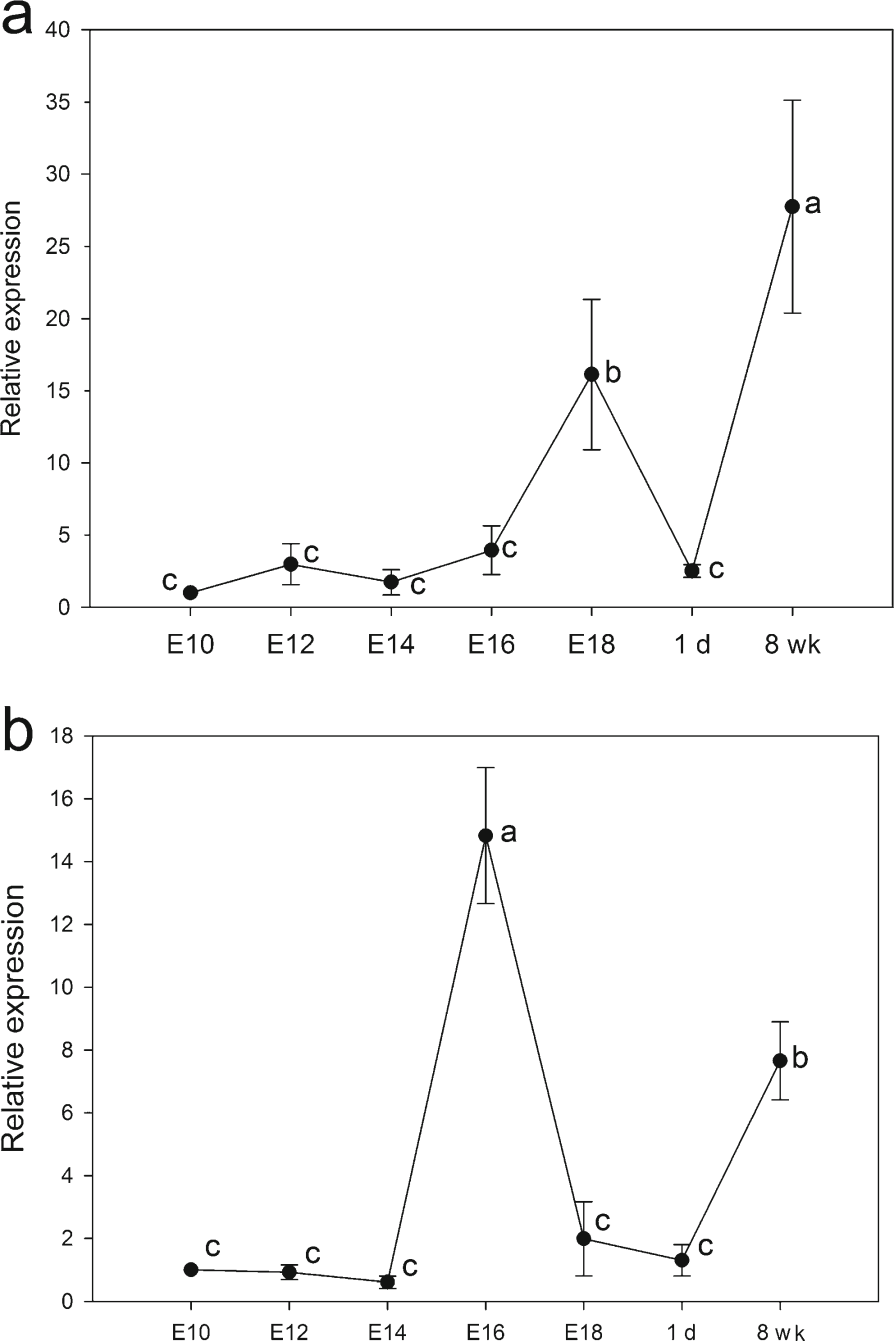
Quantitative RT-PCR analysis of *prdm1* during the development of the bursa of fabricius and spleen. cDNAs(**a**) from the bursa of fabricius on E10, E12, E14, E16 and E18 and at 1 day (*1d*) and 8 weeks (*8wk*) and (**b**) from the spleen on E10, E12, E14, E16 and E18 and at 1 day and 8 weeks were amplified with *prdm1*-specific primers. The expression level of *prdm1* was calculated relative to the expression of *gapdh* by the 2^-ΔΔCt^ method and is expressed as the fold change relative to that in the bursa of fabricius or spleen on E10. Data are expressed as the mean ± SE of five independent experiments. Values with the *same letter* do not differ significantly according to the Kruskal-Wallis test; *P* < 0.05

In order to examine the time-dependent expression of *prdm1* in the bursa of fabricius and spleen, cDNAs from these organs at various developmental stages were analyzed by qRT-PCR. The expression profiles of *prdm1* in the two organs were similar and each had two peaks during development (Fig. 4). In particular, during the development of the bursa of fabricius (Fig. 4a), *prdm1* expression was significantly elevated on E18 and then decreased at 1 day. However, the expression level of *prdm1* reached its second peak at 8 weeks. During splenic maturation (Fig. 4b), *prdm1* expression was maintained at the same level until E14 and then increased to its highest level on E16. After E16, *prdm1* expression was significantly decreased until 1 day and also reached its second peak at 8 weeks.

### Quantitative analysis of *prdm1* during development of skin and feathers

**Fig. 5:**
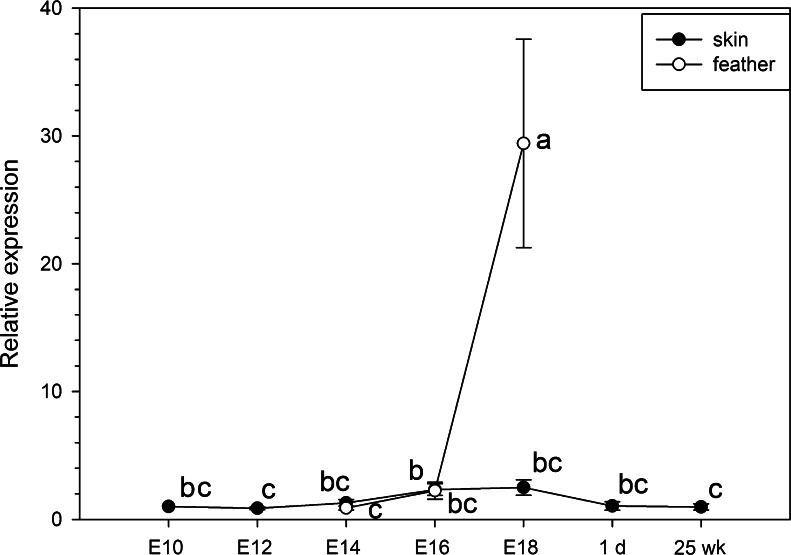
Quantitative RT-PCR analysis of *prdm1* during the development of the skin and feathers. cDNAs from the skin on E10, E12, E14, E16 and E18 and at 1 day and 25 weeks and from the feathers on E14, E16 and E18 were amplified with *prdm1*-specific primers. The expression level of *prdm1* was calculated relative to the expression of *gapdh* by the 2^-ΔΔCt^ method and is expressed the fold change relative to that in the skin on E10. Data are expressed as the mean ± SE of five independent experiments. Values with the *same letter* do not differ significantly according to the Kruskal-Wallis test; *P* < 0.05

Because our RT-PCR analysis revealed that *prdm1* was strongly expressed in the skin and feathers, we next detected the relative expression level of *prdm1* during their formation. In the skin, *prdm1* expression slightly increased from E10 to E18 and then slightly decreased at 1 day (Fig. 5). During feather formation, the level of *prdm1* mRNA significantly increased from E14 to E18 (Fig. 5).

### Quantitative analysis of *prdm1* during germline development

**Fig. 6:**
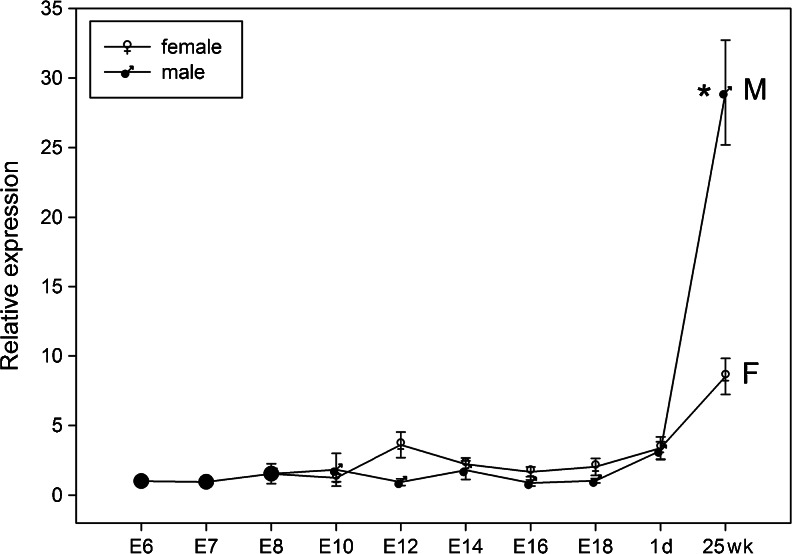
Quantitative RT-PCR analysis of *prdm1* during germline development. cDNAs from the gonads on E6, E7 and E8, from male and female gonads on E10, E12, E16 and E18 and from testes and ovaries from 1 day (*1d*) and 25 weeks (*25wk*) were amplified with *prdm1*-specific primers. The expression level of *prdm1* was calculated relative to the expression of *gapdh* by the 2^-ΔΔCt^ method and is expressed as the fold change relative to that in the gonads on E6. Data are expressed as the mean ± SE of five independent experiments. Statistically significant differences from the *prdm1* levels in the testes and ovaries on 25 weeks were analyzed by Student’s *t* test; *P < 0.05 (*M* male, *F* female)

We then examined the relative expression patterns of *prdm1* during germline development and sexual maturation by qRT-PCR. The expression of *prdm1* was continuously detected in the gonads at all the stages investigated and was significantly up-regulated in the adult (Fig. 6). Surprisingly, although a tendency was apparent for females to express a slightly higher level than males during the embryonic stage, this pattern was reversed after sexual maturation. The expression level of *prdm1* in the ovary was significantly lower than that in the testis at 25 weeks (Fig. 6).

### Expression and localization of PRDM1 in chicken tissues

**Fig. 7:**
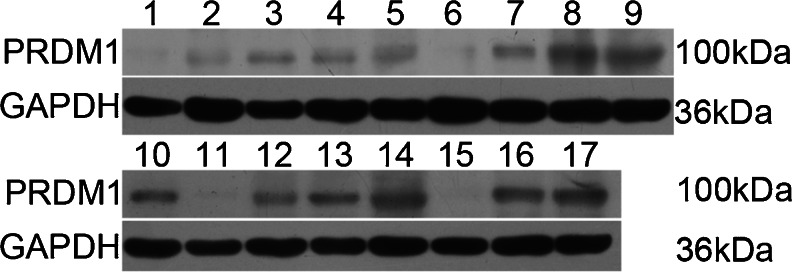
Characterization of PRDM1 expression in various chicken tissues at 1 day via Western blot analysis. GAPDH was used as a loading control. The monoclonal anti-PRDM1 antibody was used. PRDM1 was detected at ∼100 kDa (*lanes 1–17* heart, liver, skin, lung, kidney, brain, eye, bursa of fabricius, spleen, proventriculus, gizzard, intestine, testis, ovary, skeletal muscle, tongue and thymus, respectively)

To examine the expression of the PRDM1 protein, various tissues at 1 day were analyzed by immunoblotting. A single band corresponding to a 100-kDa protein was specifically detected in the same chicken tissues in which we detected the *prdm1* mRNA (Fig. 7). The observed size was in accordance with the size predicted from the *prdm1* open reading frame sequence. PRDM1 was detected in the liver, skin, lung, kidney, eye, bursa of fabricius, spleen, proventriculus, intestine, testis, ovary, tongue and thymus but was barely detected in the heart, brain, or skeletal muscle. In addition, the immunoreactive signal in the gizzard was extremely weak.

**Fig. 8:**
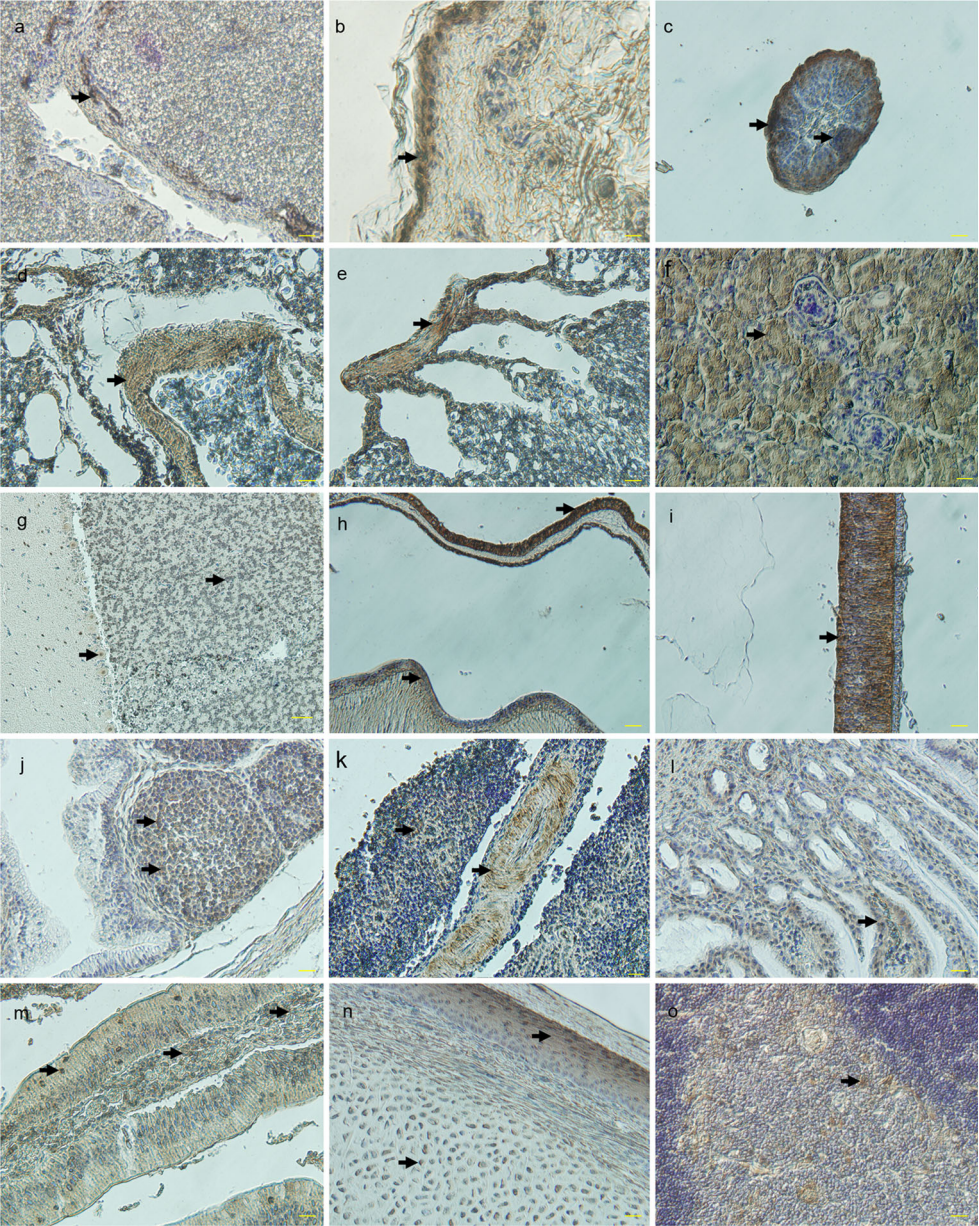
Localization of PRDM1 in various chicken tissues, as revealed by immunocytochemistry (IHC). Tissue sections were incubated with the monoclonal anti-PRDM1 antibody; hematoxylin counterstaining. PRDM1-positive cells (*arrows*) were observed in the blood vessel endothelia of the liver at 1 day (**a**), the epidermis of the skin at the adult stage (**b**), the covering sheath, barbule cells and papilla of the feather follicles on E14 (**c**), the smooth muscle of the vascular wall and parabronchus in the lung at the adult stage (**d**, **e**), the tubular structures of the kidney at the adult stage (**f**), the granule cells and Purkinje cells of the cerebellum on E14 (**g**), the corneal epithelia, lens epithelia and retina in the eye on E6 (**h**, **i**), the medulla of the bursa of fabricius at 1 day (**j**), the blood vessel endothelia and white pulp of the spleen at 1 day (**k**), the plica of the proventriculus at 1 day (**l**), the intestinal villi at the adult stage (**m**), the lingual epithelia and hyaline cartilage of the tongue at 1 day (**n**) and the medulla of the thymus at 1 day (**o**). *Bars* 20 μm (**a–f**, **h–o**), 50 μm (**g**)

**Fig. 9:**
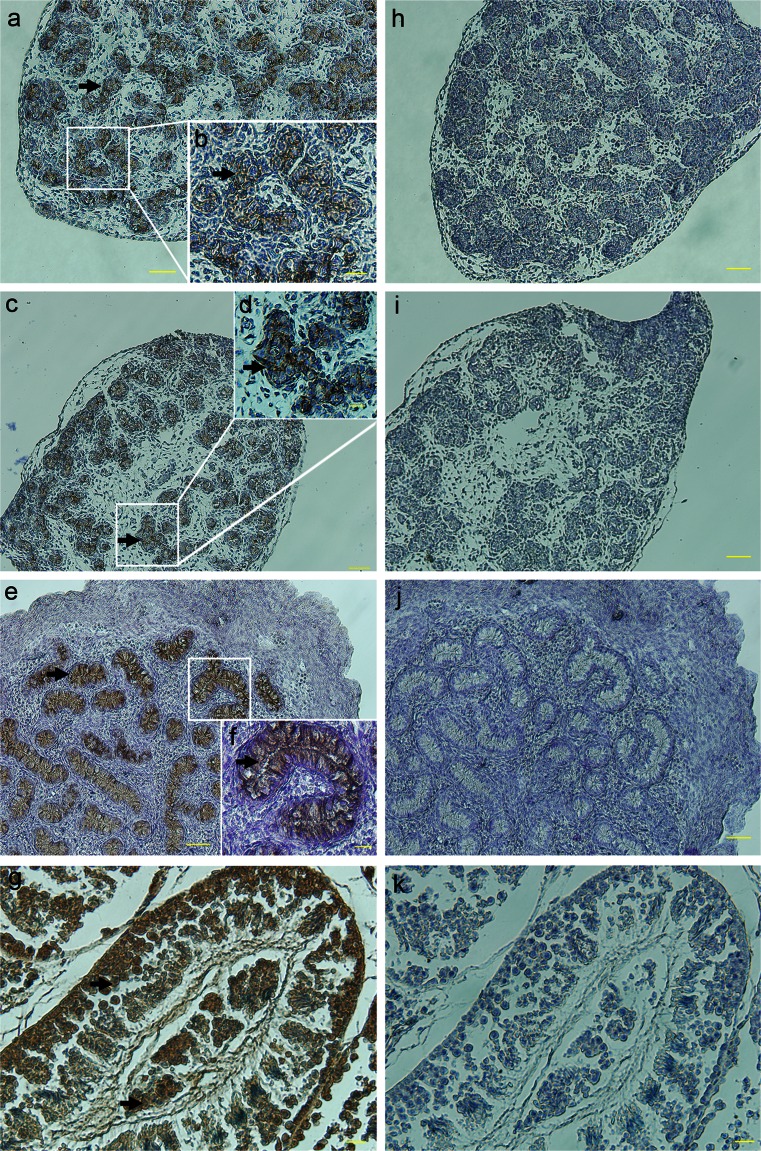
Protein localization of PRDM1 during male germline development and sexual maturation. Sections of male gonads on E14 (**a**, **b**) and testes at 1 day (**c**, **d**), 8 weeks (**e**, **f**) and 25 weeks (**g**) were analyzed by using the monoclonal anti-PRDM1 antibody. PRDM1 was continuously detected in the germ cells (*arrows*, **a–g**) and the lumen of the seminiferous tubules (*arrows*, **c–f**) of the testis. **h–k** Negative controls on E14 (**h**) and at 1 day (**i**), 8 weeks (**j**) and 25 weeks (**k**). *Bars* 50 μm (**a**, **c**, **e**, **h–j**), 20 μm (**b**, **d**, **f**, **g**, **k**)

**Fig. 10:**
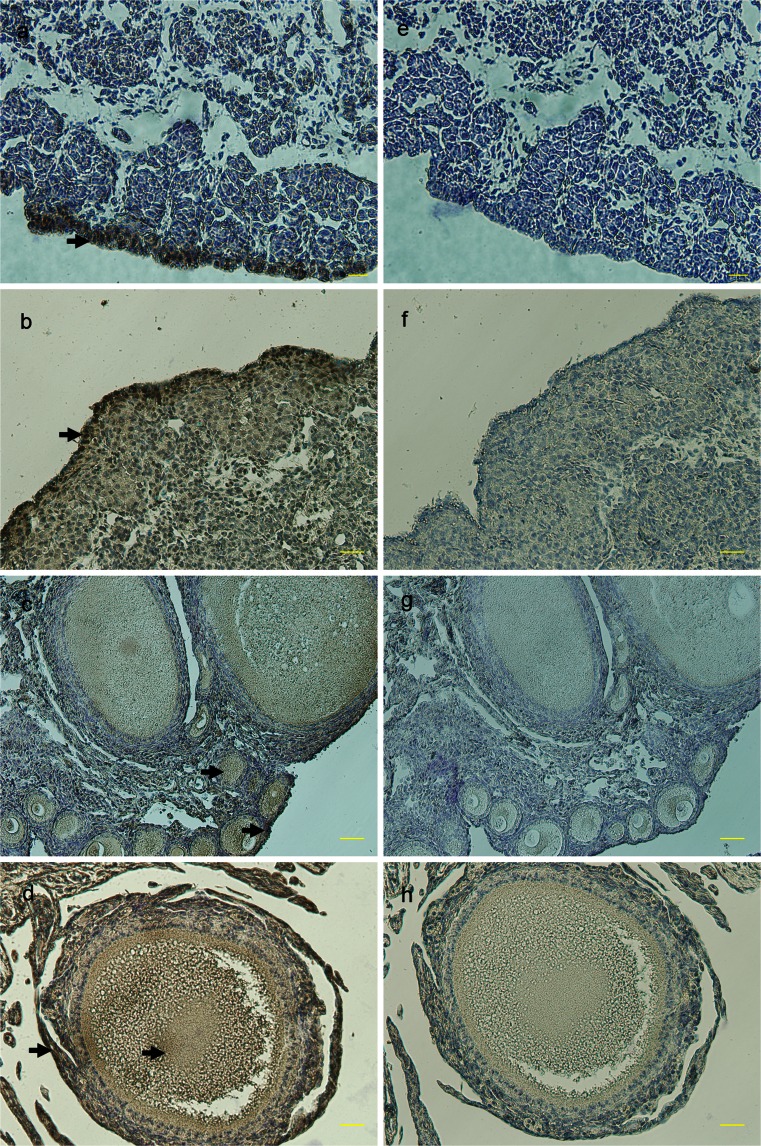
Protein localization of PRDM1 during female germline development and sexual maturation. Sections of female gonads on E14 (**a**) and ovaries at 1 day (**b**), 8 weeks (**c**) and 25 weeks (**d**) were analyzed by using the monoclonal anti-PRDM1 antibody. PRDM1 was expressed in the germinal epithelium during many stages of oogenesis but not in the medulla (*arrows*, **a–d**). PRDM1 was also weakly detected in the yolk-laden cytoplasm at 8 weeks and 25 weeks (*arrows*, **c**, **d**). **e–h** Negative controls on E14 (**e**) and at 1 day (**f**), 8 weeks (**g**) and 25 weeks (**h**). *Bars* 50 μm (**b–d**, **f-h**), 20 μm (**a**, **e**)

To examine the localization of PRDM1 further, we performed IHC by using the commercially available PRDM1 antibody. PRDM1 expression was ubiquitously apparent in various tissues (Fig. 8), except for the heart, brain, skeletal muscle and gizzard (not shown). These results generally agreed with the Western blot results, except for the gizzard. The observed negative staining of the gizzard might have been attributable to the lower abundance of PRDM1 in this tissue. Specifically, PRDM1 was mainly visible in blood vessel endothelia of the liver at 1 day (Fig. 8a), the epidermis of the skin at the adult stage (Fig. 8b), the covering sheath, barbule cells and papilla of the feather follicles on E14 (Fig. 8c), the smooth muscle of the vascular wall and parabronchus in the lung at the adult stage (Fig. 8d, e), the tubular structures of the kidney at the adult stage (Fig. 8f), the granule cells and Purkinje cells of the cerebellum on E14 (Fig. 8g), the corneal epithelia, lens epithelia and retina in the eye on E6 (Fig. 8h, i), the medulla of the bursa of fabricius at 1 day (Fig. 8j), the blood vessel endothelia and white pulp of the spleen at 1 day (Fig. 8k), the plica of the proventriculus at 1 day (Fig. 8l), the intestinal villi at the adult stage (Fig. 8m), the lingual epithelia and hyaline cartilage of the tongue at 1 day (Fig. 8n), the medulla of the thymus at 1 day (Fig. 8o) and the germ cells of the testis and ovary (Figs. 9, 10). No staining was found when any of the primary antibodies were replaced with non-immune serum (see Supplementary Fig. S1).

### Protein localization of PRDM1 during germline development and sexual maturation

We carried out further experiments on the localization of PRDM1 in the germline. Paraffin sections of male and female gonads on E14 and testes and ovaries at 1 day, 8 weeks and 25 weeks were subjected to IHC to examine the localization of PRDM1 during germline development and sexual maturation (Figs. 9, 10). In males, PRDM1 was continuously detected in the germ cell cytoplasm from E14 to 25 weeks (Fig. 9). On E14, PRDM1 was detected in the prospermatogonia (Fig. 9a, b). From 1 day to 8 weeks, PRDM1 was detected in the germ cells and the lumen of seminiferous tubules (Fig. 9c-f). At 25 weeks, cells positive for PRDM1 included those in many of the stages of spermatogenesis (Fig. 9g). However, PRDM1 was not observed within the lumen of seminiferous tubules of the mature testis (Fig. 9g). Parallel analysis of ovary sections revealed that PRDM1 was expressed in the germ cells of the ovary (Fig. 10). PRDM1 was expressed in the germinal epithelium during many stages of oogenesis but not in the medulla (Fig. 10a-d). In oocytes, except the germinal epithelium, PRDM1 was also weakly detected in the yolk-laden cytoplasm at 8 weeks and 25 weeks (Fig. 10c, d). The negative controls did not demonstrate specific staining (Figs. 9h-k, 10e-h).

### PRDM1 as a marker of germ cells in gonads and of PGCs in blood

**Fig. 11:**
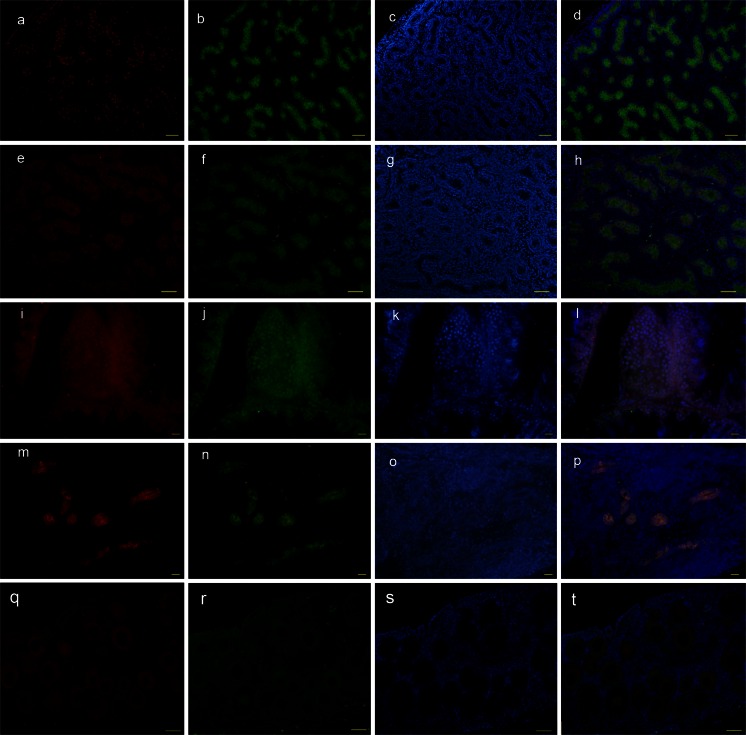
PRDM1 as a marker of germ cells in chicken gonads. The monoclonal anti-PRDM1 antibody was used. **a–d** Sections of testis at 1 day were double-stained for the chicken vasa homolog (CVH; **a**) and PRDM1 (**b**) and the nuclei were stained with DAPI (**c**). **d** Merged image of **a–c**. **e–h** Sections of testis at 1 day were double-stained for chicken deleted in azoospermia-like (cDAZL; **e**) and PRDM1 (**f**) and the nuclei were stained with DAPI (**g**). **h** Merged image of **e–g**. **i–l** Sections of testis at 25 weeks were double-stained for CVH (**i**) and PRDM1 (**j**) and the nuclei were stained with DAPI (**k**). **l** Merged image of **i–k**. **m–p** Sections of ovaries at 8 weeks were double-stained for CVH (**m**) and PRDM1 (**n**) and the nuclei were stained with DAPI (**o**). **p** Merged image of **m–o**. **q–t** Sections of ovaries at 25 weeks were double-stained for CVH (**q**) and PRDM1 (**r**) and the nuclei were stained with DAPI (**s**). **t** Merged image of **q–s**. *Bars* 50 μm (**a–h**, **q–t**), 20 μm (**i–p**)

**Fig. 12:**
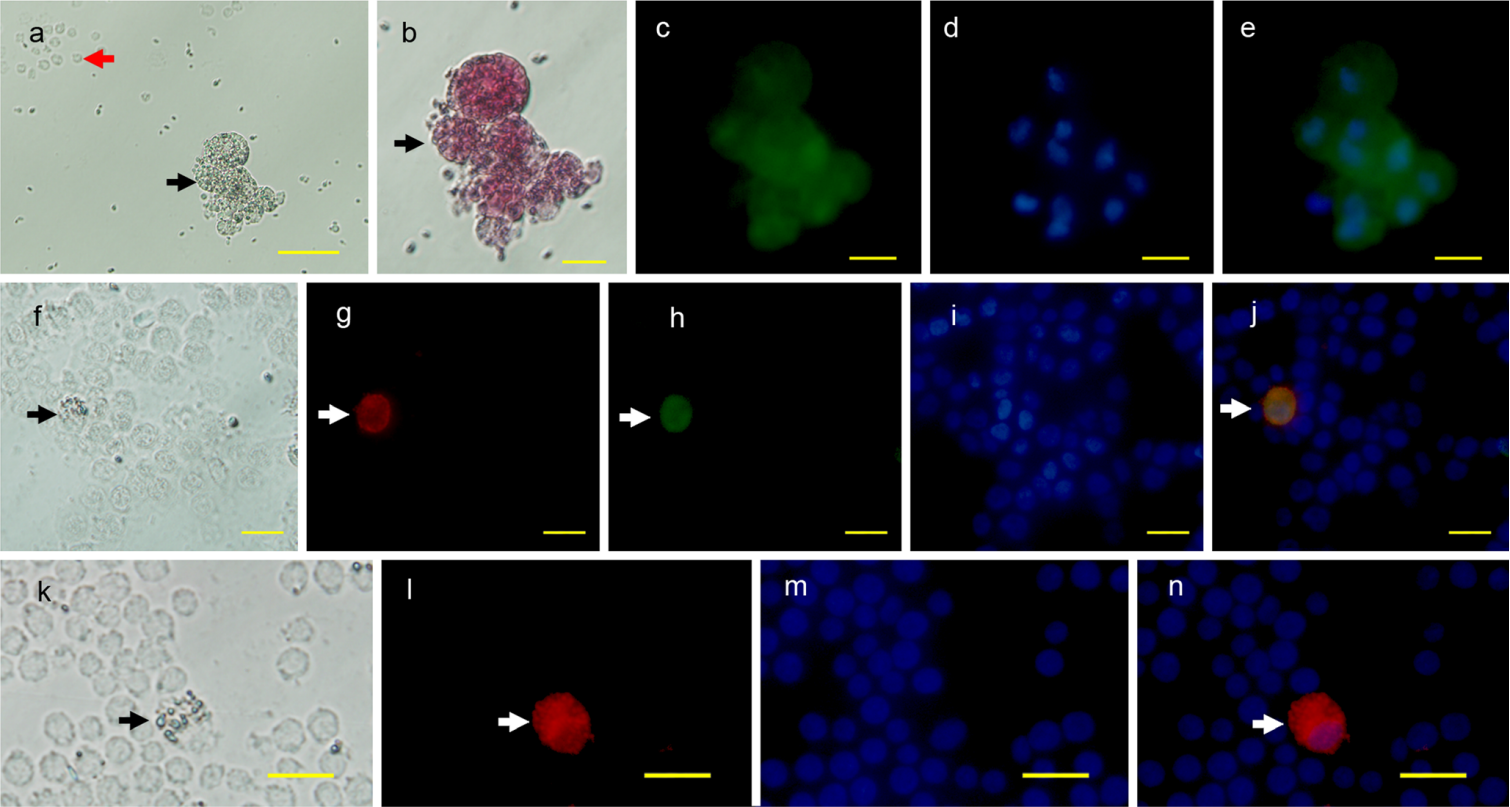
PRDM1 as a marker of circulating primordial germ cells (PGCs). Blood samples collected from chicken embryos at stages 13–15 were smeared onto glass slides. The monoclonal anti-PRDM1 antibody was used. **a–e** Cells were subjected to PRDM1 and periodic-acid-Schiff (PAS) staining. **a** Phase contrast; the circulating PGCs (*black arrow*) were round and large compared with other blood cells (*red arrow*). The large round PGCs were positive for PAS staining (**b**) and PRDM1 (**c**). **d** Nuclei stained with DAPI. **e** Merged image of **c**, **d**. **f-j** Cells were double-stained for stage-specific embryonic antigen-1 (SSEA-1; **g**, *white arrow*) and PRDM1 (**h**). **f** Phase contrast. **i** Nuclei stained with DAPI. **j** Merged image of **g–i**. **k-n** Cells were stained for CVH (**l**, *white arrow*). **k** Phase contrast. **m** Nuclei stained with DAPI. **n** Merged image of **l**, **m**. *Bars* 50 μm (**a**), 20 μm (**b–n**)

Whereas the PRDM1-positive cells in the gonads were identified as germ cells by morphology, in order to characterize these cells further, we stained them for several genes reported to be germ cell markers. CVH is an RNA helicase family protein and its expression is restricted to germline cells (Tsunekawa et al. 2000). cDAZL is a germline-specific RNA-binding protein and has also been identified in germ cells (Rengaraj et al. 2010; Saunders et al. 2003). We observed that both PRDM1 and CVH/cDAZL were co-expressed in the cytoplasm of the germ cells in the testis, in which they were distributed along the basement membrane of the seminiferous tubules (Fig. 11a-l). Double-staining with CVH/cDZAL and PRDM1 clearly revealed that PRDM1 was also present in the lumen of seminiferous tubules of the testes at 1 day (Fig. 11a-h). Notably, cDAZL was not found in the premature chicken ovary (data not shown), although CVH was present in the ovary. This is consistent with the reported localization of cDAZL in chicken ovary (Kito et al. 2010). PRDM1 and CVH were co-expressed in the early follicle (Fig. 11m-p) and adult ovary (Fig. 11q-t). PRDM1-positive cells were also identified in blood samples at stages 13–15 (Fig. 12). These cells (black arrows) were generally large and round (20.55 ± 0.72 μm in diameter), being slightly larger than those observed in previous reports of PGCs in blood (Fujimoto et al. 1976; Meyer 1964). These cells could easily be distinguished from the blood cells (red arrows) by their morphological characteristics. Moreover, SSEA-1- and CVH-immunoreactive signals and periodic-acid-Schiff (PAS) staining were also found in the PRDM1-positive cells (Fig. 12b, f-n).

### Protein localization of PRDM1 in BCs

**Fig. 13:**
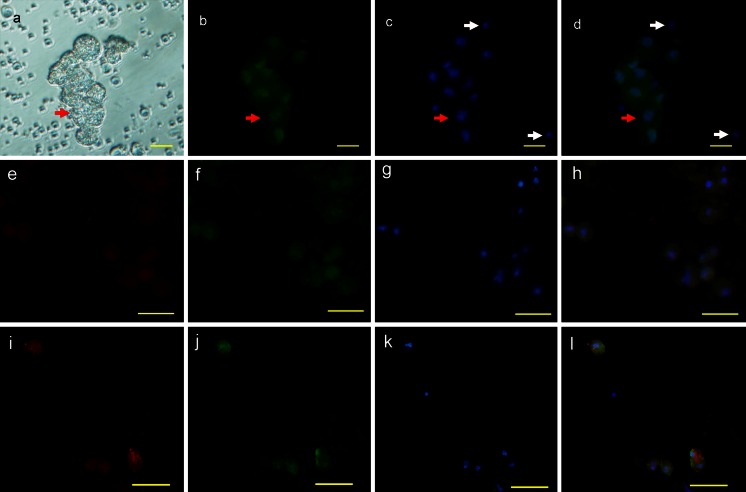
Protein localization of PRDM1 in the BCs at stage X. BCs were collected from the blastoderms at stage X and smeared onto glass slides. The monoclonal anti-PRDM1 antibody was used. **a–d** PRDM1 was localized in the nucleus and was diffused throughout the cytoplasm in some BCs (*red arrows*). PRDM1-Negative cells are indicated with *white arrows*. **a** Phase contrast. **c** Nuclei stained with DAPI. **d** Merged image of **b**, **c**. **e–h** Cells were double-stained for SSEA-1 (**e**) and PRDM1 (**f**). **g** Nuclei stained with DAPI. **h** Merged image of **e–g**. **i–l** Cells were double-stained for CVH (**i**) and PRDM1 (**j**). **k** Nuclei stained with DAPI. **l** Merged image of **i–k**. *Bars* 20 μm (**a–d**), 50 μm (**e–l**)

We focused our final experiments on the localization of PRDM1 in early BCs. Immunostaining with an anti-PRDM1 antibody displayed strong nuclear localization and diffuse staining throughout the cytoplasm in some BCs (red arrows, Fig. 13a-d). Negative cells are indicated with white arrows in Fig. 13a-d. The positive cells were large (20.55 ± 0.68 μm in diameter) and had a large spherical nuclei, which corroborates previous reports of CVH-positive cells (∼ 20 μm in diameter; Tsunekawa et al. 2000) and SSEA-1-positive cells (20.82 ± 0.86 μm in diameter) in the blastoderm (Karagenc et al. 1996). To test whether these PRDM1-positive cells in the blastoderm were presumptive PGCs, we then stained them with an anti-SSEA-1 or anti-CVH antibody. Double-staining revealed that both PRDM1 and CVH/SSEA-1 were co-expressed in some BCs (Fig. 13e-l).

## Discussion

PRDM1 is a highly conserved and evolutionarily ancient protein that regulates the development of many tissues in a variety of organisms. However, a wealth of data supports the notion that PRDM1 shows strong context dependency (Shaffer et al. 2002). In the present study, we investigated the spatio-temporal expression patterns of PRDM1 in various tissues, especially in the germline, during chicken development. Below, we will compare the expression pattern of PRDM1 between chickens and other organisms and will speculate on the possible roles of this protein in the chicken.

As many of the results of this work rest on the specificity of the antibody for chicken PRDM1, we presented several lines of evidence that support the specificity of PRDM1 staining. First, two antibodies designed against different epitopes have been used and give similar results (the results of the polyclonal PRDM1 antibody are not shown). Next, for our Western blot analysis, the antibody recognizes a band of 100 kDa, which is the predicted size of chicken PRDM1. Finally, the mRNA results and protein results are similar.

The lymphocyte-related functions of PRDM1 have been extensively characterized in mice and humans (Chang et al. 2000; Kallies et al. 2006; Shaffer et al. 2002; Shapiro-Shelef et al. 2003). However, little is known about PRDM1 in the spleen and bursa of fabricius, in which B cell development takes place, in the chicken. Here, qRT-PCR revealed that the expression profiles of *prdm1* in the bursa of fabricius and spleen are similar and have two peaks during development (Fig. 4). PRDM1-postive cells were located in the medulla of the bursa of fabricius and are diffusely scattered throughout the spleen (Fig. 8j, k). Based on our observations, we propose that PRDM1 has a role in the organogenesis of the spleen and bursa of fabricius in the chicken.

In mouse, PRDM1 expression in the foregut is restricted to a subset of endodermal epithelial cells at the headfold stage but is not observed in its derivatives, such as lungs, liver and thymus (Chang and Calame 2002). However, in the present study, PRDM1 expression was detected in these tissues. IHC revealed PRDM1 in the smooth muscle of the vascular wall and parabronchus in the lung (Fig. 8d, e), the blood vessel endothelia of the liver (Fig. 8a) and the medulla of the thymus (Fig. 8o). Interestingly, in mice, PRDM1 mutant embryos lack the thymus, although this is because the loss of PRDM1 disrupts caudal pharyngeal morphogenesis; the thymus normally forms from an outpocketing of the pharyngeal endoderm of the third arch (Robertson et al. 2007). Additionally, transient PRDM1 expression also precisely identifies the progenitors of the anterior or second heart field on E9.5 (Robertson et al. 2007). However, in our study, PRDM1 expression has not been observed in chicken heart.

In the digestive system, PRDM1 is strongly expressed throughout the epithelium of the embryonic gut and regulates postnatal reprogramming of the intestinal enterocytes in mouse (Harper et al. 2011). Similarly, we observed PRDM1 expression in the intestine of chick. PRDM1 was also localized in the plica of the proventriculus (Fig. 8l) but not in the gizzard. During tongue development, PRDM1 is expressed in the lingual epithelium and hyaline cartilage (Fig. 8n), consistent with previous observations in mouse (Chang and Calame 2002). In the urinary system, PRDM1 was detected in the tubular structures of the developing kidney (Fig. 8f), which has not been described in mouse.

PRDM1 is also strongly expressed in skin epithelial cell lineages, including the suprabasal layer, upper granular layer, dermal papillae, inner hair shaft and sebaceous gland (Horsley et al. 2006; Magnusdottir et al. 2007). PRDM1 defines sebocyte stem cells via the repression of c-MYC (a nuclear oncogene) expression (Horsley et al. 2006) and is also an important regulator of epidermal terminal differentiation in mice (Magnusdottir et al. 2007). Recently, PRDM1 has been identified as a marker of terminal differentiation but not of sebocytic progenitor cells (Sellheyer and Krahl 2010). In our study, qRT-PCR revealed that *prdm1* is persistently and strongly expressed in the skin and that it is slightly decreased in adults (Fig. 5). Notably, during feather formation, the relative level of *prdm1* is significantly increased on E18 (Fig. 5). This change might be related to cornification, the final differentiative step for epidermal keratinocytes, which involves dramatic cell condensation before death (Candi et al. 2005). In feathers on E18, the quantity of *gapdh* mRNA is significantly decreased. However, the quantity of *prdm1* mRNA is not significantly changed. IHC has revealed PRDM1 in the epidermis of the skin and in the covering sheath, barbule cells and papilla of the feather follicles (Fig. 8b, c). In addition, during skin development, BMPs induce dermal markers and ectopic feather tracts in the chicken (Scaal et al. 2002). Considering our data and the following reports showing that PRDM1 is induced by BMPs in PGCs (Ohinata et al. 2005) and represses c-MYC in sebocyte differentiation in mice, that c-MYC transcripts are restricted to the highly proliferating epidermis in chickens (Desbiens et al. 1991) and that the morphogenesis of skin appendages is driven by a series of reciprocal epithelial-mesenchymal interactions between the prospective epidermis and the dermal mesenchyme, we suggest that PRDM1 is associated with the formation of the skin and feathers. In neural-ectoderm-derived tissues, PRDM1 expression has been reported in the primitive photoreceptors of the neural retina (Chang and Calame 2002; Wilm and Solnica-Krezel 2005) and the disruption of PRDM1 in both zebrafish and mouse leads to a loss of photoreceptor cells. However, PRDM1 is not expressed in the brain or other neural-ectoderm-derived tissues in mouse (Chang and Calame 2002). In chicken, PRDM1 mRNA has also been detected in the developing eyes (Ha and Riddle 2003). However, no expression of PRDM1 in the neural crest of chicks has been reported, although the embryonic stages examined might possibly have been too late for such observations. Here, we have shown, by RT-PCR and Western blot analysis, that PRDM1 is indeed expressed in the eye. IHC evidence further suggests that PRDM1 is localized in the corneal epithelia, lens epithelia and retina in the eye on E6 (Fig. 8h, i). We did not detect PRDM1 in the chicken brain in this study; however, interestingly, it was detected in the granule cells and Purkinje cells of the cerebellum by IHC.

In zebrafish, PRDM1 promotes the formation of slow myosin heavy chain (MyHC)-expressing myocytes by repressing the fast program during development (Baxendale et al. 2004). In contrast, no requirement for PRDM1 activation of the slow muscle program has been found in the mouse myotome, although it is expressed in the first differentiating myocytes of the early myotome (Vincent et al. 2012). In the chick embryo, PRDM1 is expressed in all these different types of myogenic cells (Beermann et al. 2010). However, in the present study, our RT-PCR, immunoblotting and IHC studies have unexpectedly shown that PRDM1 is not expressed in the skeletal muscle or myocardium but rather in the smooth muscle, such as the vascular walls and parabronchus in the lung and the blood vessel endothelia in the spleen (Fig. 8d, e, k), contradicting the report of Beermann et al. (2010). This disparity might be attributable to differences of sample stages and regions. Moreover, whether PRDM1 becomes limited to the posterior limb bud regions, as suggested by the previous study of chicken embryos (Ha and Riddle 2003), remains to be determined. The role of PRDM1 during the formation of smooth muscle should therefore be investigated in further experiments.

Where and when does the germline arise in animals? Data from model organisms show that germ cells can be specified either by maternally inherited determinants (preformation) or inductive signals (epigenesis; Extavour and Akam 2003). Here, PRDM1 expression has been observed in presumptive PGCs at stage X and in PGCs in the blood and germ cells in gonads. At stage X, the PRDM1-postive cells are large and SSEA-1/CVH-positive (Fig. 13e-l); 25–45 SSEA-1-positive cells or approximately 30 CVH-positive cells are presumptive PGCs at stage X (Karagenc et al. 1996; Tsunekawa et al. 2000). Therefore, we speculate that at least some of the PRDM1-positve cells are presumptive PGCs. Our qRT-PCR results from early embryonic samples revealed that *prdm1* expression significantly changes from stage V to stage 18 (∼ 84 h; Fig. 3) indicating that *prdm1* expression is precisely regulated by a temporal program. However, unfortunately, we did not detect the expression of *prdm1* at stage I (uterine age of 0–1 h), the first cleavage. Therefore, we do not know whether *prdm1* is expressed at a high level at the first cleavage. The origin of avian PGCs remains speculative and merits further study. Interestingly, germ cell specification in zebrafish is predetermined by the inheritance of germ plasm and PRDM1 is not expressed in zebrafish PGCs (Ng et al. 2006).

PRDM1-positive cells were also observed in blood at stages 13–15. These cells show PGC-like morphological characteristics and are positive for SSEA-1, CVH and PAS staining (Fig. 12). SSEA-1 is an appropriate marker for germ cells in chicken embryos older than stage 10, because the only SSEA-1-reactive cells in avian embryos beyond stage 10 are PGCs (Mozdziak et al. 2005). PAS is the classic histological stain for differentiating PGCs from somatic cells (Meyer 1960). Based on the results described here, we infer that these PRDM1-positive cells are PGCs in blood and that PRDM1 is a marker of PGCs at this stage. Staining with this marker might thus be a novel method of choice for the characterization of chicken PGCs.

In gonads, analysis of the co-localization of PRDM1 and CVH/cDAZL has demonstrated PRDM1 continuously in the cytoplasm of germ cells until the adult stage (Figs. 9, 10). This finding differs from a previous report in mice in which PRDM1 expression in PGCs has been found to be rapidly terminated after E13 (Chang and Calame 2002). qRT-PCR has revealed that *prdm1* is continuously expressed in the gonads at all the stages investigated and is significantly up-regulated in both male and female adults (Fig. 6). Moreover, the level of *prdm1* mRNA in the testis is significantly higher than that in the ovary at the adult stage. In addition, IHC/IF revealed that PRDM1 is also present in the lumen of seminiferous tubules from E14 to 8 weeks (Figs. 9a-f, 11a-h) but disappears in adults (Figs. 9g, 11i-l). These findings suggest that the expression of chicken PRDM1 is differentially controlled depending on the sex and stage of sexual maturation. Taking these data together, we infer that PRDM1 expression is conserved in PGCs and during germline differentiation until the adult stage, making it a novel molecular marker for studies of PGC differentiation and germ cell development in the chicken.

Notably, SSEA-1 is a marker of pluripotency (Solter and Knowles 1978) and is expressed in undifferentiated cells, including chick embryonic stem cells (cESCs) derived from BCs at stage X (Pain et al. 1996). Cells derived from the chick blastoderm at stage X are heterogeneous. Some of these cells are pluripotent and contribute to both the somatic and germinal tissue when injected into recipient embryos leading to the formation of chimeras (Petitte et al. 1990; Thoraval et al. 1994). Therefore, are PRDM1-positive cells at stage X also heterogeneous and are some of them pluripotent? In mice, PRDM1 has been shown to repress the somatic program in PGCs (Ohinata et al. 2005). However, a recent study has clearly demonstrated that PRDM1 is dispensable for the derivation and maintenance of pluripotent stem cells and reprogramming and that the repression of somatic gene expression by PRDM1 is a PGC-specific phenomenon (Bao et al. 2012). Indeed, PRDM1 is down-regulated during the reprogramming of normal PGCs to give pluripotent embryonic germ cells, which are equivalent to ESCs, suggesting that PRDM1 is critical for the maintenance of germ cells, although it might restrict reversion to a pluripotent state (Durcova-Hills et al. 2008; Leitch et al. 2010). In chicken, initial BCs present germline competency but their culture in vitro leads to a progressive loss of this property (van de Lavoir et al. 2006b). Only PGCs maintained under specific non-adherent culture conditions have been demonstrated to retain this property (van de Lavoir et al. 2006a). The exogenous expression of CVH results in the down-regulation of cPOUV and cNANOG and induces cESC reprogramming toward a germ cell fate, thereby showing the fine balance between the level of expression of pluripotency-associated genes and germline-specific genes (Lavial et al. 2009). Thus, the testing of whether the modulation of PRDM1 expression in cESCs will change their behavior should be of interest. Further experiments on the role of PRDM1 in cESCs and PGCs should help to answer the question of which model, the predetermination model or the inductive model, is valid in the chick.

Although we clearly describe the expression pattern of PRDM1 in the chicken, our study is limited and further experiments are needed to determine the potential role of PRDM1 in the chicken. For example, viable PRDM1-postive cells can be sorted from heterogeneous BCs and then their gene expression profile can be analyzed. These cells can subsequently be injected into recipient embryos to produce chimeras and to trace their fate.

In summary, PRDM1 mRNA and protein were found to be expressed in a wide range of chicken tissues and are continuously expressed in the germline. The expression pattern of PRDM1 suggests that it plays an important role in chicken embryonic and germline development and also reflects the differences in the evolution of PRDM1 between birds and mammals. This study also demonstrated that, in addition to PAS and an antibody against SSEA-1, chicken PGCs can be recognized by a PRDM1 antibody. However, functional studies are needed to elucidate the actual contribution of PRDM1 within chicken PGCs.

## Electronic supplementary material

Below is the link to the electronic supplementary material.

ESM 1 (JPEG 237 kb)

High Resolution Image (TIFF 14004 kb)
