# Supplementary figures and images for: Expression patterns of prdm1 during chicken embryonic and germline development
Source: Cell Tissue Res. 2014 Apr 2;356(2):341–56. doi: 10.1007/s00441-014-1804-1 (PMC4015062; doi:10.1007/s00441-014-1804-1)

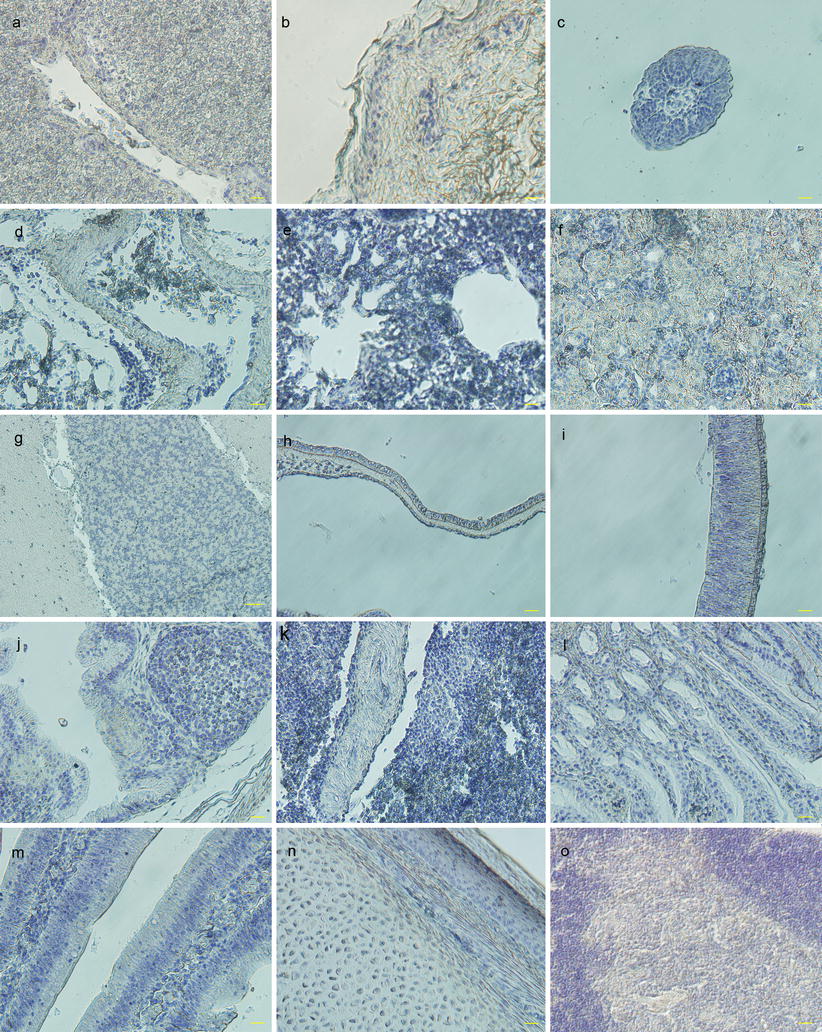

Supplement: Supplementary file 1 — (JPEG 237 kb) [file 441_2014_1804_Fig14_ESM.jpg]

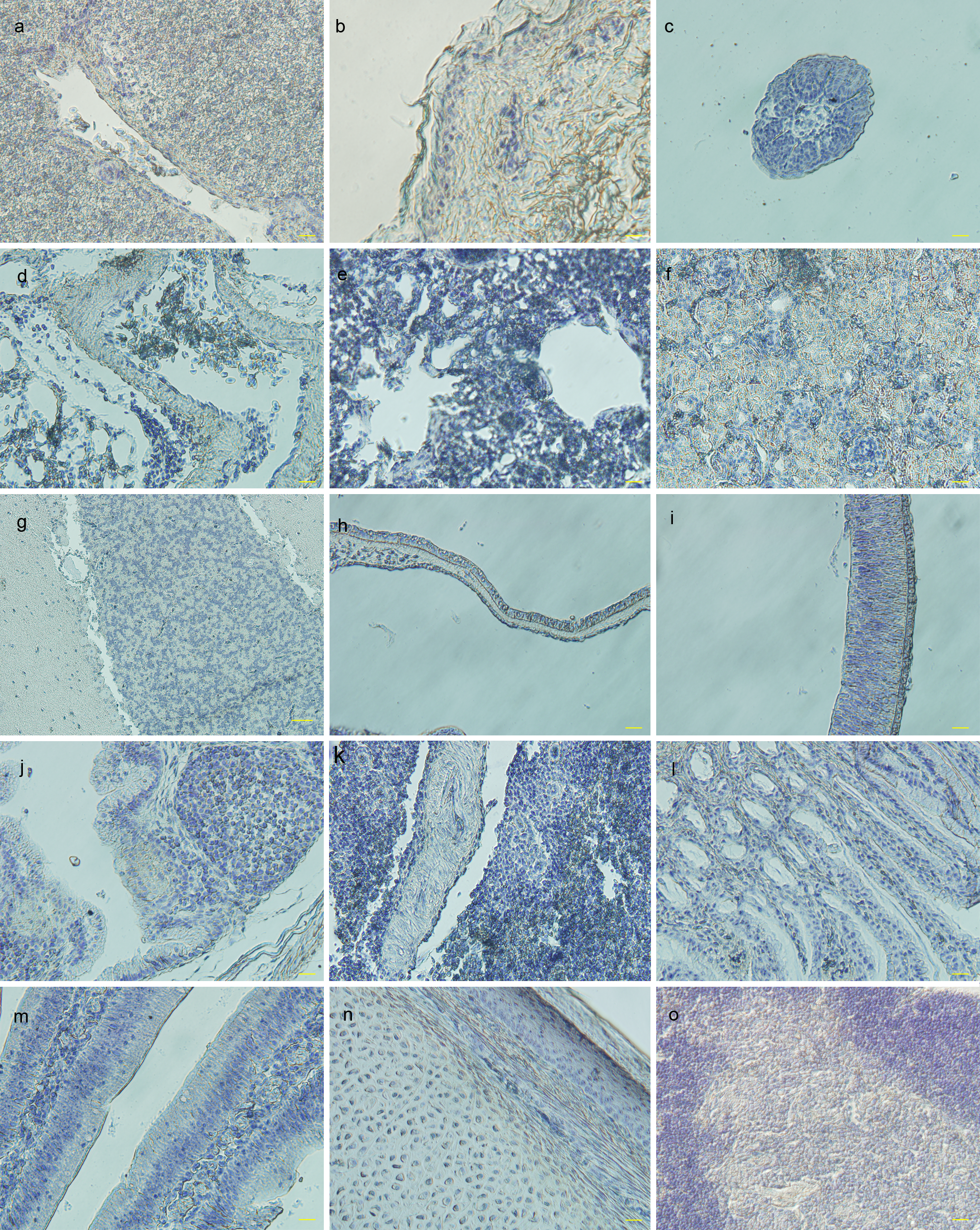

Supplement: Supplementary file 2 — High Resolution Image (TIFF 14004 kb) [file 441_2014_1804_MOESM1_ESM.tif]
